# 2-Chloro-*N*-(3,5-dichloro­phen­yl)acetamide

**DOI:** 10.1107/S1600536808000366

**Published:** 2008-01-11

**Authors:** B. Thimme Gowda, Sabine Foro, Hartmut Fuess

**Affiliations:** a Department of Chemistry, Mangalore University, Mangalagangotri 574 199, Mangalore, India; b Institute of Materials Science, Darmstadt University of Technology, Petersenstrasse 23, D-64287, Darmstadt, Germany

## Abstract

The structure of the title compound, C_8_H_6_Cl_3_NO, is closely related to that of *N*-(3,5-dichloro­phen­yl)acetamide and other amides. The mol­ecular skeleton is essentially planar. The mol­ecules in the crystal structure are stabilized by N—H⋯O and N—H⋯Cl inter­molecular hydrogen bonds running along the *a* axis

## Related literature

For related literature, see: Gowda *et al.* (2007 ▶, 2007*a* ▶,*b* ▶); Shilpa & Gowda (2007 ▶).
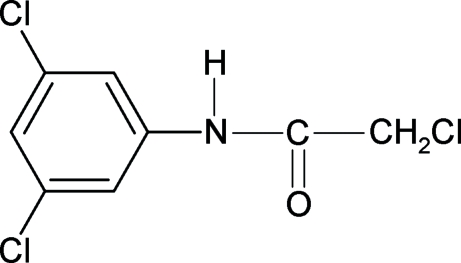


## Experimental

### 

#### Crystal data

C_8_H_6_Cl_3_NO
*M* *_r_* = 238.49
Monoclinic, 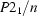

*a* = 4.567 (1) Å
*b* = 24.350 (4) Å
*c* = 8.903 (2) Å
β = 102.20 (2)°
*V* = 967.7 (3) Å^3^
*Z* = 4
Cu *K*α radiation
μ = 8.23 mm^−1^
*T* = 299 (2) K
0.60 × 0.35 × 0.13 mm

#### Data collection

Enraf–Nonius CAD-4 diffractometer
Absorption correction: ψ scan (North *et al.*, 1968 ▶) *T*_min_ = 0.063, *T*_max_ = 0.354
3732 measured reflections
1730 independent reflections
1606 reflections with *I* > 2σ(*I*)
*R*_int_ = 0.073
3 standard reflections frequency: 120 min intensity decay: 1.0%

#### Refinement

*R*[*F* ^2^ > 2σ(*F* ^2^)] = 0.098
*wR*(*F* ^2^) = 0.288
*S* = 1.39
1730 reflections
118 parameters
H-atom parameters constrained
Δρ_max_ = 0.57 e Å^−3^
Δρ_min_ = −1.12 e Å^−3^

### 

Data collection: *CAD-4-PC* (Enraf–Nonius, 1996 ▶); cell refinement: *CAD-4-PC*; data reduction: *REDU4* (Stoe & Cie, 1987 ▶); program(s) used to solve structure: *SHELXS97* (Sheldrick, 2008 ▶); program(s) used to refine structure: *SHELXL97* (Sheldrick, 2008 ▶); molecular graphics: *PLATON* (Spek, 2003 ▶); software used to prepare material for publication: *SHELXL97*.

## Supplementary Material

Crystal structure: contains datablocks I, global. DOI: 10.1107/S1600536808000366/om2202sup1.cif

Structure factors: contains datablocks I. DOI: 10.1107/S1600536808000366/om2202Isup2.hkl

Additional supplementary materials:  crystallographic information; 3D view; checkCIF report

## Figures and Tables

**Table 1:** Hydrogen-bond geometry (Å, °)

*D*—H⋯*A*	*D*—H	H⋯*A*	*D*⋯*A*	*D*—H⋯*A*
N1—H1N⋯O1^i^	0.86	2.37	3.019 (4)	133
N1—H1N⋯Cl3^i^	0.86	2.68	3.482 (3)	156

Symmetry code: (i) 
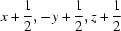
.

**Fig. 1.**
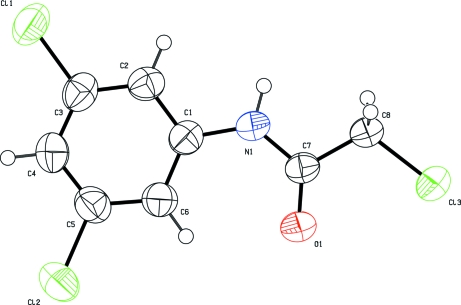
Molecular structure of the title compound, showing the atom labeling scheme. The displacement ellipsoids are drawn at the 50% probability level.

**Fig. 2.**
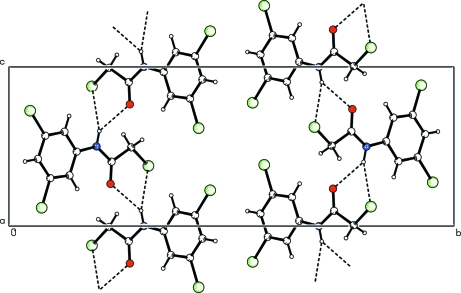
Molecular packing of the title compound with hydrogen bonding shown as dashed lines.

## supplementary crystallographic information

### Comment

In the present work, the structure of 2-chloro-*N*-(3,5-dichlorophenyl)- acetamide (35DCPCA) has been determined to study the effect of substituents on the structures of *N*-aromatic amides (Gowda *et al.*, 2007*a*, *b*; Gowda *et al.*, 2007). The structure of 35DCPCA (Fig. 1) is closely related to 2-chloro-*N*-(2-chlorophenyl)acetamide (2CPCA), 2-chloro-*N*-(4-chlorophenyl)acetamide (4CPCA)(Gowda *et al.*, 2007*b*), *N*-(3,5-dichlorophenyl)-acetamide (35DCPA) (Gowda *et al.*, 2007*a*) and other amides (Gowda *et al.*, 2007). The molecular skeleton is essentially planar. The bond parameters in 35DCPCA are similar to those in 2CPCA, 4CPCA, 35DCPA and other acetanilides (Gowda *et al.*, 2007*a*, *b*; Gowda *et al.*, 2007). The simultaneous intermolecular N—H···O and N—H···Cl hydrogen bonds (Table 1) link the molecules into chains running along the *a* axis (Fig. 2).

### Experimental

The title compound was prepared according to the literature method (Shilpa & Gowda, 2007). The purity of the compound was checked by determining its melting point. It was characterized by recording its infrared and NMR spectra (Shilpa & Gowda, 2007). Single crystals of the title compound were obtained from an ethanolic solution and used for X-ray diffraction studies at room temperature.

### Refinement

The CH atoms were positioned with idealized geometry using a riding model with C—H = 0.93–0.97 Å. The NH atom was located in difference map with N—H = 0.86 Å. *U*_iso_(H) values were set equal to 1.2 *U*_eq_ of the parent atom.

### Crystal data

**Table:**

C_8_H_6_Cl_3_NO	*F*_000_ = 480
*M**_r_* = 238.49	*D*_x_ = 1.637 Mg m^−^^3^
Monoclinic, *P*2_1_/*n*	Cu *K*α radiation λ = 1.54180 Å
Hall symbol: -P 2yn	Cell parameters from 25 reflections
*a* = 4.567 (1) Å	θ = 6.2–23.2º
*b* = 24.350 (4) Å	µ = 8.23 mm^−^^1^
*c* = 8.903 (2) Å	*T* = 299 (2) K
β = 102.20 (2)º	Long plate, colourless
*V* = 967.7 (3) Å^3^	0.60 × 0.35 × 0.13 mm
*Z* = 4	

### Data collection

**Table:**

Enraf–Nonius CAD-4 diffractometer	*R*_int_ = 0.074
Radiation source: fine-focus sealed tube	θ_max_ = 66.9º
Monochromator: graphite	θ_min_ = 3.6º
*T* = 299(2) K	*h* = 0→5
ω/2θ scans	*k* = −29→23
Absorption correction: ψ scan(North *et al.*, 1968)	*l* = −10→10
*T*_min_ = 0.063, *T*_max_ = 0.354	3 standard reflections
3732 measured reflections	every 120 min
1730 independent reflections	intensity decay: 1.0%
1606 reflections with *I* > 2σ(*I*)	

### Refinement

**Table:**

Refinement on *F*^2^	Secondary atom site location: difference Fourier map
Least-squares matrix: full	Hydrogen site location: inferred from neighbouring sites
*R*[*F*^2^ > 2σ(*F*^2^)] = 0.098	H-atom parameters constrained
*wR*(*F*^2^) = 0.288	*w* = 1/[σ^2^(*F*_o_^2^) + (0.2*P*)^2^] where *P* = (*F*_o_^2^ + 2*F*_c_^2^)/3
*S* = 1.39	(Δ/σ)_max_ = 0.005
1730 reflections	Δρ_max_ = 0.57 e Å^−^^3^
118 parameters	Δρ_min_ = −1.12 e Å^−^^3^
Primary atom site location: structure-invariant direct methods	Extinction correction: none

### Special details

**Table:**

Geometry. All e.s.d.'s (except the e.s.d. in the dihedral angle between two l.s. planes) are estimated using the full covariance matrix. The cell e.s.d.'s are taken into account individually in the estimation of e.s.d.'s in distances, angles and torsion angles; correlations between e.s.d.'s in cell parameters are only used when they are defined by crystal symmetry. An approximate (isotropic) treatment of cell e.s.d.'s is used for estimating e.s.d.'s involving l.s. planes.
Refinement. Refinement of *F*^2^ against ALL reflections. The weighted *R*-factor *wR* and goodness of fit *S* are based on *F*^2^, conventional *R*-factors *R* are based on *F*, with *F* set to zero for negative *F*^2^. The threshold expression of *F*^2^ > σ(*F*^2^) is used only for calculating *R*-factors(gt) *etc*. and is not relevant to the choice of reflections for refinement. *R*-factors based on *F*^2^ are statistically about twice as large as those based on *F*, and *R*- factors based on ALL data will be even larger.

### Fractional atomic coordinates and isotropic or equivalent isotropic displacement parameters (Å^2^)

**Table:**

	*x*	*y*	*z*	*U*_iso_*/*U*_eq_	
C1	0.7994 (8)	0.15227 (14)	0.4676 (4)	0.0509 (8)	
C2	0.9691 (7)	0.12475 (16)	0.5925 (4)	0.0528 (9)	
H2	0.9617	0.1353	0.6921	0.063*	
C3	1.1486 (9)	0.08169 (15)	0.5677 (5)	0.0585 (10)	
C4	1.1657 (10)	0.06515 (16)	0.4216 (5)	0.0639 (10)	
H4	1.2884	0.0362	0.4059	0.077*	
C5	0.9960 (10)	0.09289 (17)	0.3011 (5)	0.0615 (10)	
C6	0.8060 (8)	0.13604 (15)	0.3183 (4)	0.0553 (9)	
H6	0.6881	0.1533	0.2336	0.066*	
C7	0.4446 (7)	0.23062 (14)	0.4049 (4)	0.0466 (8)	
C8	0.3181 (8)	0.27475 (16)	0.4947 (4)	0.0539 (9)	
H8A	0.4787	0.2992	0.5425	0.065*	
H8B	0.2388	0.2575	0.5758	0.065*	
N1	0.6266 (7)	0.19666 (13)	0.5008 (3)	0.0513 (8)	
H1N	0.6394	0.2030	0.5970	0.062*	
O1	0.3874 (5)	0.22828 (12)	0.2651 (3)	0.0560 (8)	
Cl1	1.3597 (3)	0.04771 (4)	0.72484 (15)	0.0797 (6)	
Cl2	1.0161 (4)	0.07364 (6)	0.11529 (14)	0.0948 (6)	
Cl3	0.0336 (2)	0.31342 (4)	0.37802 (10)	0.0626 (5)	

### Atomic displacement parameters (Å^2^)

**Table:**

	*U*^11^	*U*^22^	*U*^33^	*U*^12^	*U*^13^	*U*^23^
C1	0.0514 (16)	0.0467 (17)	0.0520 (18)	−0.0028 (13)	0.0048 (14)	0.0006 (14)
C2	0.0540 (19)	0.0512 (18)	0.0503 (18)	−0.0072 (14)	0.0042 (14)	−0.0012 (14)
C3	0.057 (2)	0.0451 (17)	0.068 (2)	−0.0029 (14)	−0.0001 (17)	0.0021 (16)
C4	0.073 (2)	0.0481 (17)	0.071 (2)	0.0086 (17)	0.0157 (19)	−0.0015 (18)
C5	0.073 (2)	0.055 (2)	0.059 (2)	−0.0001 (16)	0.0189 (17)	−0.0056 (16)
C6	0.064 (2)	0.0501 (19)	0.051 (2)	−0.0011 (15)	0.0115 (16)	−0.0001 (15)
C7	0.0435 (15)	0.0520 (17)	0.0421 (16)	−0.0029 (13)	0.0038 (12)	0.0003 (13)
C8	0.0540 (18)	0.062 (2)	0.0406 (17)	0.0106 (15)	−0.0004 (13)	−0.0021 (14)
N1	0.0544 (15)	0.0573 (16)	0.0382 (14)	0.0062 (12)	0.0010 (12)	−0.0012 (12)
O1	0.0566 (14)	0.0654 (16)	0.0413 (13)	0.0070 (11)	−0.0002 (10)	−0.0017 (11)
Cl1	0.0871 (9)	0.0616 (8)	0.0782 (9)	0.0179 (5)	−0.0100 (7)	0.0057 (5)
Cl2	0.1422 (14)	0.0816 (10)	0.0661 (9)	0.0296 (7)	0.0342 (8)	−0.0070 (6)
Cl3	0.0580 (7)	0.0717 (8)	0.0538 (8)	0.0174 (4)	0.0023 (5)	0.0058 (4)

### Geometric parameters (Å, °)

**Table:**

C1—C2	1.387 (5)	C5—Cl2	1.740 (4)
C1—C6	1.393 (5)	C6—H6	0.9300
C1—N1	1.406 (5)	C7—O1	1.218 (4)
C2—C3	1.377 (5)	C7—N1	1.343 (5)
C2—H2	0.9300	C7—C8	1.524 (5)
C3—C4	1.380 (6)	C8—Cl3	1.756 (3)
C3—Cl1	1.732 (4)	C8—H8A	0.9700
C4—C5	1.363 (6)	C8—H8B	0.9700
C4—H4	0.9300	N1—H1N	0.8600
C5—C6	1.392 (5)		
			
C2—C1—C6	120.5 (3)	C5—C6—C1	117.3 (3)
C2—C1—N1	116.5 (3)	C5—C6—H6	121.3
C6—C1—N1	123.0 (3)	C1—C6—H6	121.3
C3—C2—C1	119.3 (3)	O1—C7—N1	126.2 (3)
C3—C2—H2	120.3	O1—C7—C8	123.1 (3)
C1—C2—H2	120.3	N1—C7—C8	110.7 (3)
C2—C3—C4	121.9 (3)	C7—C8—Cl3	112.5 (2)
C2—C3—Cl1	118.9 (3)	C7—C8—H8A	109.1
C4—C3—Cl1	119.3 (3)	Cl3—C8—H8A	109.1
C5—C4—C3	117.5 (4)	C7—C8—H8B	109.1
C5—C4—H4	121.3	Cl3—C8—H8B	109.1
C3—C4—H4	121.3	H8A—C8—H8B	107.8
C4—C5—C6	123.5 (3)	C7—N1—C1	129.7 (3)
C4—C5—Cl2	118.6 (3)	C7—N1—H1N	115.1
C6—C5—Cl2	117.9 (3)	C1—N1—H1N	115.1
			
C6—C1—C2—C3	1.1 (5)	Cl2—C5—C6—C1	−177.9 (3)
N1—C1—C2—C3	−178.5 (3)	C2—C1—C6—C5	−2.2 (5)
C1—C2—C3—C4	0.3 (6)	N1—C1—C6—C5	177.4 (3)
C1—C2—C3—Cl1	179.9 (3)	O1—C7—C8—Cl3	10.7 (5)
C2—C3—C4—C5	−0.4 (6)	N1—C7—C8—Cl3	−170.4 (3)
Cl1—C3—C4—C5	179.9 (3)	O1—C7—N1—C1	2.0 (6)
C3—C4—C5—C6	−0.9 (6)	C8—C7—N1—C1	−176.8 (3)
C3—C4—C5—Cl2	179.2 (3)	C2—C1—N1—C7	179.5 (3)
C4—C5—C6—C1	2.1 (6)	C6—C1—N1—C7	0.0 (6)

### Hydrogen-bond geometry (Å, °)

**Table:**

*D*—H···*A*	*D*—H	H···*A*	*D*···*A*	*D*—H···*A*
N1—H1N···O1^i^	0.86	2.37	3.019 (4)	133
N1—H1N···Cl3^i^	0.86	2.68	3.482 (3)	156

Symmetry codes: (i) *x*+1/2, −*y*+1/2, *z*+1/2.
